# Optimized detection of circulating anti-nuclear envelope autoantibodies by immunofluorescence

**DOI:** 10.1186/1471-2172-7-20

**Published:** 2006-09-06

**Authors:** Vagia Tsiakalou, Elena Tsangaridou, Hara Polioudaki, Artemissia-Phoebe Nifli, Meri Koulentaki, Tonia Akoumianaki, Elias Kouroumalis, Elias Castanas, Panayiotis A Theodoropoulos

**Affiliations:** 1 Biochemistry, University of Crete, School of Medicine, P.O. Box 2208, Heraklion 71003, Greece; 2 Experimental Endocrinology, University of Crete, School of Medicine, P.O. Box 2208, Heraklion 71003, Greece; 3 Gastroenterology, University of Crete, School of Medicine, P.O. Box 2208, Heraklion 71003, Greece

## Abstract

**Background:**

Antinuclear antibodies are useful diagnostic tools in several autoimmune diseases. However, the routine detection of nuclear envelope autoantibodies using immunofluorescence (IF) is not always easy to perform in patients' sera because of the presence of autoantibodies to other nuclear and cytoplasmic components which could mask the characteristic rim-like pattern of nuclear envelope autoantibodies. This is particularly common in sera from patients with primary biliary cirrhosis (PBC), which generaly have high titres of anti-mitochondrial antibodies. Therefore, we have assayed a number of commercial slides and alternative fixation conditions to optimize the detection of anti-nuclear envelope antibodies (ANEA) in PBC sera.

**Methods:**

We have explored the presence of ANEA in 33 sera from patients with established PBC using three different Hep2 commercial slides and home-made slides with HeLa and Hep2 cells fixed with methanol, ethanol, 1% or 4% formaldehyde.

**Results:**

We observed that the IF pattern was related to the cell type used (Hep2 or HeLa), the manufacturer and the cell fixation scheme. When both cell lines were fixed with 1% formaldehyde, the intensity of the cytoplasmic staining was considerably decreased regardless to the serum sample, whereas the prevalence of cytoplasmic autoantibodies was significantly lowered, as compared to any of the Hep2 commercial slide and fixation used. In addition, the prevalence of ANEA was importantly increased in formaldehyde-fixed cells.

**Conclusion:**

Immunofluorescence using appropriately fixed cells represent an easy, no time-consuming and low cost technique for the routine screening of sera for ANEA. Detection of ANEA is shown to be more efficient using formaldehyde-fixed cells instead of commercially available Hep2 cells.

## Background

Circulating antinuclear antibodies (ANA) is a diverse group of autoantibodies found in patients with systemic or organ specific autoimmune diseases and a variety of infections, but also in asymptomatic normal individuals, although in low titres. ANA screening helps establishing diagnosis in patients with clinical features suggestive of an autoimmune or connective tissue disorders, while excluding (at least partially) the possibility of an autoimmune disorder in patients with few or uncertain clinical findings. In addition, they assist in monitoring disease progression and activity. Nuclear envelope is a complex structure consisting of outer and inner nuclear membranes, nuclear pore complexes (NPC) and the nuclear lamina [1]. Autoantibodies against nuclear envelope proteins exhibit a characteristic rim-like/peripheral pattern in IF. In a number of diseases, such as chronic fatigue syndrome, primary biliary cirrhosis and lupus or lupus-like syndrome the detection of anti-nuclear envelope antibodies may give an additional diagnostic clue [2-5]. In chronic fatigue syndrome, [6] appr. 52% of patients develop autoantibodies to components of the nuclear envelope, mainly nuclear lamins. In systemic lupus erythematosus [5] a strong association of autoantibodies to human nuclear lamin B1 with lupus anticoagulant antibodies (LAC) has been reported and suggested that the presence of LAC without anti-lamin B1 may define a subset of SLE patients at greater risk for thrombosis.

In PBC, a chronic destructive cholangitis, although ninety percent of patients show circulating antimitochondrial antibodies (AMA) [7], the presence of ANA is highly specific [8] and can be used as a "positive tool" in the diagnosis of AMA-negative PBC cases [9]. Indeed, 64% of sera from PBC patients were found positive for ANA [10]. Several proteins have been recognized as ANA targets in PBC, such as Sp100 [11] and promyelocytic leukemia proteins [12], the latter generating a multiple nuclear dot pattern in IF. Antibodies against proteins of the nuclear pore complex, such as gp210 and p62, have been reported [13,14], being associated with the activity and severity of the disease [3]. In addition, it was recently suggested that anti-p62 antibodies may be related to the progressive or advanced state of PBC [4]. In rare cases (1–2%) antibodies against lamin B receptor, an integral protein of the inner nuclear membrane may be found [8]. Using standard IF methods, the prevalence of ANEA in PBC, differs considerably amidst references, varying between 29% and 58% [4,13,15,16]. This discrepancy may be due to the use of different commercially available slides, or to the simultaneous presence of other autoantibodies in patient's sera, directed against different nucleoplasmic or cytoplasmic antigens, possibly masking perinuclear staining.

In the present work we propose an alternative protocol, using formaldehyde-fixed HeLa or Hep2 cells, to improve recognition of nuclear envelope proteins by circulating autoantibodies. The specificity of autoantibodies against nuclear envelope antigens was also confirmed by immunoblotting using purified HeLa nuclei and nuclear envelopes. Finally, the IF analysis of 33 sera from patients with established PBC, using formaldehyde-fixed cells and three commercially available slides showed that ANEA are more accurately detected in formaldehyde-fixed cells than in commercial slides.

## Methods

### Patients and sera

Frozen (-80°C) serum samples from thirty three patients (twenty nine women) with PBC were used. Median age at the time of venesection was 60 years (ranging from 32 to 75 years). All patients had histologically proven PBC, 16 were at stage I-II, 6 at stage III and 11 at stage IV. Patients were followed at the Department of Gastoenterology, University Hospital of Heraklion, Greece, were on ursodeoxycholic acid (15 mg/kg) sinse diagnosis, no one had ever been on steroids or other immunomodulatory treatment and no one had undergone liver transplantation. Oral informed concent from all patients participating in this study was taken, to use their blood samples for research purposes. Ethical approval was issued by the Scientific and Ethics Committee of the University Hospital of Heraklion.

### Cell lines and culture

Hep2 cells (ECACC 86030501) were found to have a HeLa profile by DNA fingerprinting and therefore recently re-designated as "HeLa derivative" cells. Hep2 (larynx and cervical carcinoma) cells were obtained from the European Collection of Cell Cultures (ECACC, Salisbury, Wiltshire) and cervical carcinoma HeLa cells (CCL-2), from the American Type Tissue Culture Collection (Manassas, VA). Both cell lines were maintained at 37°C, in a humidified atmosphere containing 5% CO_2 _and cultured in Dulbecco's MEM or EMEM for HeLa and Hep2, respectively (Biochrom, Berlin, Germany), supplemented with 10% heat-inactivated fetal bovine serum, penicillin and streptomycin.

### Indirect immunofluorescence microscopy

Indirect immunofluorescence was performed as described previously [17]. Briefly, Hep2 and HeLa cells grown on coverslips were washed with PBS, fixed in 4% or 1% formaldehyde for 5 minutes at room temperature or in cold (-20°C) methanol or ethanol for 5 minutes and permeabilized with Triton X-100. Fixed cells were incubated in blocking buffer (phosphate buffered saline, pH 7.4, 0.5% Triton X-100 and 1% fish skin gelatin) and then with sera at a dilution of 1/80 in blocking buffer for 1 hour. The specimens were stained with fluorescein conjugated anti-human IgG, diluted and ready for use (Inova Diagnostics, San Diego, CA). The same sera (dilution 1/80) were examined for the presence of ANA using fixed Hep2 cells from 3 different suppliers: Inova Diagnostics (San Diego, CA), Zeus Scientific Inc. (Raritan, NJ) and bmd (Marne La Vallée, France). In all cases, detection was made by a supplied FITC-coupled anti-human Ig antibody. In all cases, we have followed the protocols suggested by the manufacturer. Fluorescence was routinely assayed in a Leica SP confocal microscope and a standard fluorescence microscope, with comparable results.

### Isolation of cell nuclei and nuclear envelopes

To isolate nuclei and nuclear envelopes, HeLa cells were detached from the culture dishes, centrifuged at 300 *g *and washed three times with phosphate buffered saline pH 7.4, containing 1 mM PMSF. The pellet was resuspended in an equal volume of ice-cold buffer H (10 mM Hepes-KOH pH 7.4, 2 mM MgCl2, 0.1 mM EGTA, 1 mM DTT, 1 mM PMSF and 2 μg/ml each of leupeptin, pepstatin and aprotinin) and Dounce-homogenized under careful phase monitoring. The homogenate was centrifuged at 1200 *g *for 10 minutes, yielding a pellet (nuclei) and a supernatant (cytoplasmic fraction). The nuclear fraction was washed in buffer H, containing 150 mM NaCl and was stored at -80°C until use.

Nuclear envelopes were prepared as described previously [18]. Briefly, nuclei were solubilised in 20 mM Tris buffer pH 8.5, containing 0.1 mM MgCl_2_, 0.5 mM PMSF, 1 mM DTT, 10% sucrose and incubated with 2 mg/ml DNAse I at room temperature for 15 minutes. DNAse digestion was repeated in Tris buffer pH 7.5. The insoluble material representing nuclear envelopes was separated by centrifugation and solubilised sequentially in high salt (2 M KCl) buffer, secondly in 40 mM Tris-HCl pH 8.0, 0.1 mM EGTA, 2 mM MgCl_2_, 1 mM DTT, 1 mM PMSF and finally in ice-cold distilled H_2_O, in order to extract most of the peripheraly attached material.

### Immunoblotting

Thirty μg of HeLa nuclei or nuclear envelopes were electrophoresed on a 12.5% SDS polyacrylamide gel and transfered on nitrocellulose membrane. Immobilized autoantigens were subjected to a denaturation/renaturation process, in order to obtain correctly folded autoepitopes and therefore optimize autoantibody reactivity. Briefly, nitrocellulose membranes were first incubated for 5 minutes in 50 mM Tris-HCl pH 8.0, containing 20% propanol-2 and washed 3 times with H_2_0. Thereafter, proteins were initially denatured by incubating membrane in 7 M Guanidine-HCl, 50 mM Tris-HCl pH 8.0, 2 mM EDTA pH 8.0, 5 mM DTT and then renatured in washing buffer (20 mM Tris-HCl pH 7.4, 155 mM NaCl, 0.1% Tween) for 10 minutes. Free sites on the membrane were blocked in 20 mM Tris-HCl pH 7.4, 155 mM NaCl, 0.1% Tween, 1% fish skin gelatin overnight at 4°C. Membranes were incubated with sera at 1/300 dilution in blocking buffer for 1 hour at room temperature and washed 3 times with the same buffer. Autoantibodies were recognized using anti-human secondary antibodies conjugated with HRP at a dilution of 1/10.000 in blocking buffer and detected using the ECL system (Amersham-Pharmacia, Buckinghamshire, UK).

### Statistical analysis

Comparison of two values was made by calculating the z-value and reporting to the normal distribution tables. Comparison of percentages (probabilities) was made by the calculation of χ^2 ^score.

## Results

### Anti-nuclear envelope antibodies are specifically detected using formaldehyde-fixed HeLa cells

**Figure 1:**
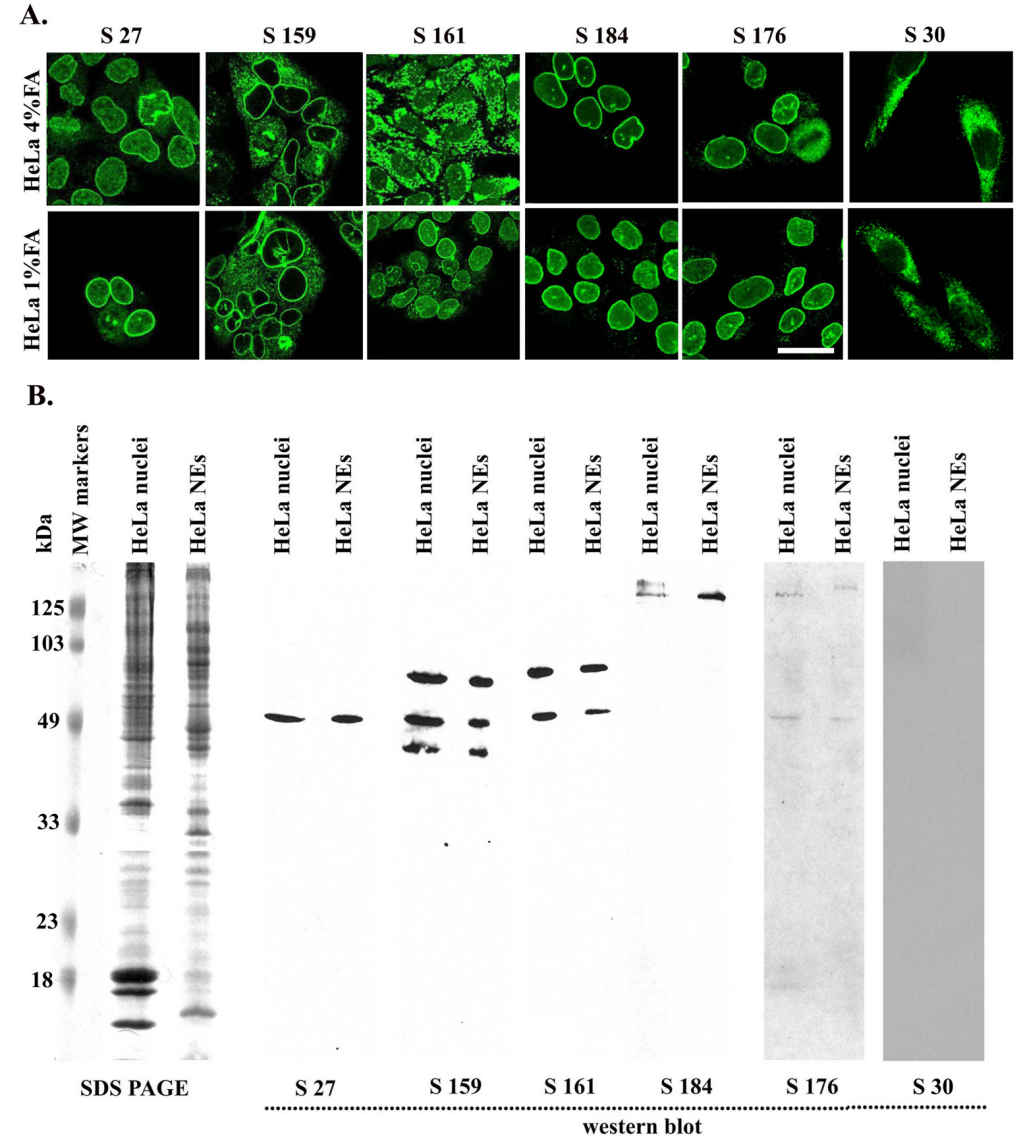
(A) Immunostaining of HeLa cells fixed with 1% or 4% formaldehyde with PBC sera diluted 1/80, Bar = 20 μm. (B) Immunobloting depicting electrophoretically fractionated nuclei and nuclear envelopes (NEs) from HeLa cells that have been probed with serum 27 (s27), serum 159 (s159), serum 161 (s161), serum 184 (s184), serum 176 (s176) or serum 30 (s30) at a dilution 1/300. At the left molecular mass markers at 125, 103, 49, 33, 23 and 18 kDa are indicated.

PBC is among autoimmune diseases in which ANEA are usually detected. In the present study, we tested sera from patients with diagnosed PBC; representative cytoplasmic and nuclear envelope IF patterns are depicted in Figure 1. HeLa cells cultured on coverslips and fixed either with 4% or 1% formaldehyde were used. When cells were fixed with 4% FA a peripheral nuclear pattern indicative of nuclear envelope staining was observed, with the exception of sera 30 and 161, which presented an intense cytoplasmic staining and a non-conclusive nuclear envelope pattern. However, fixation with 1% FA allowed the detection of nuclear envelope autoantibodies in serum 161 (Figure 1A).

In order to confirm the results obtained with IF (Figure 1A), we assayed detection of autoantigens by immunoblotting using purified nuclei and nuclear envelopes from HeLa cells. Depending on their molecular weight, nuclear envelope autoantigens recognized by autoantibodies present in the sera examined could be grouped in two categories (Figure 1B): The first comprises autoantigens of high molecular mass, recognized by sera 176 and 184, while the second contains a protein of approximately 50 kDa recognized by all sera (27, 159, 161). Two additional proteins with a molecular mass of ~90 kDa and 40 kDa were recognized by serum 159, while serum 161 recognized only the former. No signal was detected upon blotting of the afore-mentioned nuclear extracts with a serum containing anti-mitochondrial antibodies but not ANEA as verified by IF (Figure 1, s30). Therefore, we concluded that HeLa cells, appropriately fixed with FA, are suitable substrates for the specific detection of ANEA in patients' sera.

### Differential detection of anti-nuclear envelope antibodies by formaldehyde-fixed cells and commercially available Hep2 slides

Three different commercial slides/kits of Hep2 cells and home-made slides of Hep2 and HeLa cells were used for the detection of autoantibodies in patients' sera. The rationale behind the use of these two cell types is that they are both epithelial in origin (larynx and cervical carcinoma for Hep2 and cervical carcinoma for HeLa), and express the same genotype and a similar phenotype.

**Figure 2:**
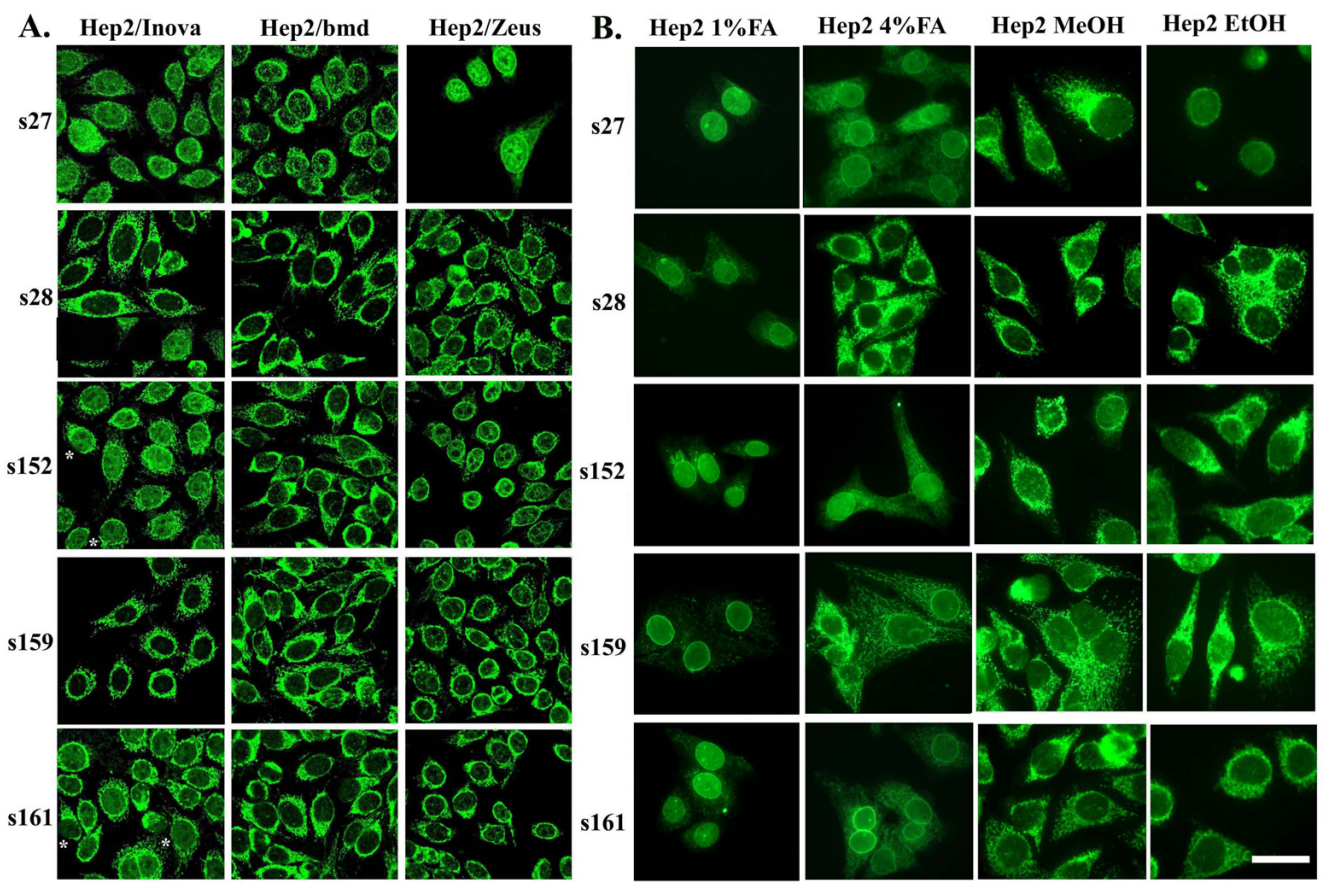
Immunostaining of Hep2 cells with PBC sera. Commercially available slides with Hep2 cells (A) and normally growing Hep2 cells (B) fixed with methanol, ethanol, 1% or 4% formaldehyde were stained with sera (s) 27, 28, 152, 159 and 161 diluted 1/80, Bar = 20 μm.

Characteristic immunofluorescence profiles of three commercially available Hep2 slides and home-made slides of Hep2 cells fixed with 1% or 4% formaldehyde, methanol or ethanol are shown in Figure 2. In Figure 2A, typical staining of sera using commercially available Hep2 slides, is depicted. Although all sera exhibited a cytoplasmic staining, its intensity varied depending on the substrate used. In general, weaker cytoplasmic staining was observed when slides from Zeus company were used. In Hep2 slides from Inova, with the exception of serum 27, no conclusive staining of the nuclear envelope was found: the perinuclear pattern obtained with sera 152 and 161 was discontinuous in some cells (asterisks), indicating a rather cytoplasmic staining, while sera 28 and 159 did not show an accentuation of the peripheral staining, suggesting failure of nuclear envelope antigen recognition. A discontinuous perinuclear staining was observed using slides from bmd, whereas nuclear envelope pattern was revealed in 3 sera (27, 152 and 159) on Zeus slides.

Analysis of the same sera using home-made slides of Hep2 cells resulted in different IF patterns depending on the fixation conditions. Hep2 cells fixed with methanol or ethanol and to a lesser extent with 4% formaldehyde showed an IF profile very similar to those obtained with commercially available slides (compare Figure 2A and 2B). The IF pattern was quite different when sera were tested on Hep2 cells fixed with 1% formaldehyde. As a rule, cytoplasmic staining was considerably lowered or eliminated thus revealing a nuclear envelope pattern (Figure 2).

**Figure 3:**
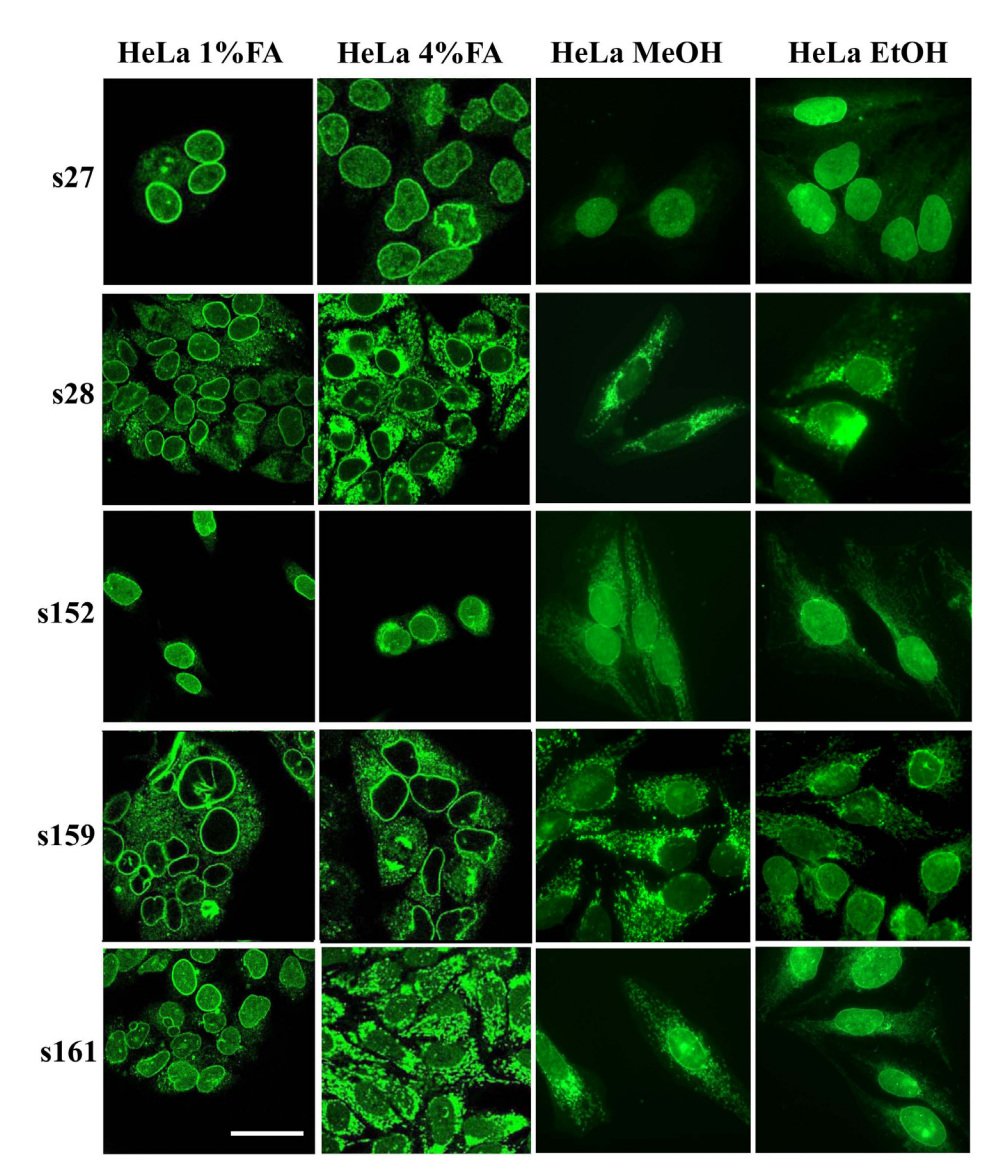
Immunostaining of HeLa cells with PBC sera. Normally growing HeLa cells fixed with methanol, ethanol, 1% or 4% formaldehyde were stained with sera (s) 27, 28, 152, 159 and 161 diluted 1/80, Bar = 20 μm.

We have further assayed the same sera using HeLa cells, a cell line widely used in experimental studies (Figure 3). As shown for Hep2 cells, when HeLa cells were fixed with 1% formaldehyde, cytoplasmic staining was considerably reduced and nuclear envelope was revealed as the predominant stained compartment (compare Figure 2B and Figure 3).

Extending our analysis in all 33 sera samples from patients with PBC, usually enriched in anti-mitochondrial antibodies, we showed that the detection of different groups of autoantibodies with IF varied considerably, depending on the provider, the fixation conditions and the cell type (Hep2, HeLa) used (Table 1). Moreover, many of the examined sera presented both cytoplasmic nuclear and/or nuclear envelope staining, while, a significant number of sera recognised solely nucleoplasmic or nuclear envelope antigens, depending on the substrate used. Concerning the detection of cytoplasmic autoantibodies, the three commercial tests and home-made slides with cells fixed with 4%FA revealed a number of positive cases, ranging from 51.5% to 75.7 %. In contrast, when Hep2 or HeLa cells fixed with 1%FA were used, a significantly lower number of positive cases, 30.3% and 27.2%, respectively were detected (p < 0.05 at least by χ^2 ^analysis, as compared to the mean of the other tests). Although nucleoplasmic staining seemed favoured in Zeus test (positiveness was 54.5%) as compared to other substrates, (ranging from 30.3% to 45.4%) no statistically significant difference was found. Finally, HeLa and Hep2 cells, fixed with either 1% or 4% FA were more efficient in the detection of ANEA, as compared to Inova and bmd kits, while Zeus kit showed an equally higher number of ANEA positive cases (21.1%).

**Table 1:** Prevalence of cytoplasmic, nucleoplasmic and NE antibodies by immunofluorescence using various substrates and fixation schemes in 33 PBC patients' sera.

Cell type/fixation	autoantigen localization					
	
	Cytoplasmic		nucleoplasmic		NE	
	
	No.	%	No.	%	No.	%
HeLa/4%FA	19	57.5	10	30.3	11	33.3
HeLa/1%FA	9	27.2	12	36.4	13	39.4
Hep2/4%FA	25	75.7	13	39.4	11	33.3
Hep2/1%FA	10	30.3	15	45.4	15	45.4
Hep2 Inova	18	54.5	13	39.4	5	15.1
Hep2 bmd	19	57.5	12	36.4	4	12.1
Hep2 Zeus	17	51.5	18	54.5	7	21.2

## Discussion

The detection of ANA by IF is routinely used for the diagnosis of autoimmune diseases. In spite of a relative facility, parameters such as the IF substrates (tissue or cell lines), the fixation conditions, the absence of a common standard and the diagnostic ability of the examiner, hinder the comparison of results performed in different laboratories. In addition, the presence of a variety of autoantibodies, directed against diverse cytoplasmic or nuclear antigens may obscur the detection of a specific class of autoantibodies with potential diagnostic relevance in a given disease. Such cases are difficult to resolve and a number of alternative methods are subsequently applied, in order to identify the target of circulating autoantibodies.

Usually, the detection of ANEA is based on the characteristic perinuclear rim-like fluorescence. Nevertheless, as shown in Figures 2 and 3, in some cases, this pattern may be masked by the presence of autoantibodies directed against other cytoplasmic or nucleoplasmic elements. In the present study, we report that formaldehyde-fixed cells are more adequate to reveal the presence of ANEA, compared to commercially available Hep2 cell substrates (Table 1). It is known that antibody reactivity could be modulated by the type of fixation. Within this study, we showed that both normal (4%) and mild (1%) formaldehyde fixation are essential to detect the totality of ANEA, since specific autoantigens were recognized only under a particular fixation condition. In most cases, mild fixation of cells with 1% formaldehyde reduced their cytoplasmic staining. It is likely that 1% FA may not adequately fix mitochondrial antigens, thus unmasking nuclear envelope structures stained by nuclear envelope autoantibodies. In addition we showed that the NE pattern obtained in 1% FA fixed cells is specific and irrelevant to autoantigen mislocalisation. Indeed, immunoblot analysis of nuclear and nuclear envelope fraction with sera, exhibiting perinuclear staining revealed the presence of specific nuclear envelope autoantigens. The proteins of high molecular mass recognized by autoantibodies of sera 176 and 184 correspond probably to gp210 antigen of the NPC. The rest of autoantigens detected by immunobloting with an apparent molecular mass of 90 kDa, 50 kDa and 40 kDa could be components of the NPC, not yet characterized. Recently, anti-NPC antibodies against proteins with similar molecular mass (86 kDa, 54 kDa and 39 kDa) were identified in PBC sera using the same method, with frequencies of 29%, 30% and 18%, respectively [4].

Besides serum titration, IF guides further diagnostic procedures, eg ELISA for specific autoantibodies or dot blot assays, relying on the fluorescence pattern. However, in sera with a mixture of autoantibodies, the pattern for ANEA in IF is not always evident and easy to confirm, especially when AMA are detected in a high titre. In fact, results reported in various studies show an under- or an over-estimation of ANEA [4,13,15,16]. The same is also true for a number of other classes of autoantibodies. Indeed, analysis of PBC sera has shown that IF is less sensitive in detecting circulating autoantibodies than immunoblotting and enzymatic immunoassay [3,16]. More than 70% of the patients with PBC, who were AMA-negative by IF, were found AMA-positive using recombinant autoantigens in a newly developed ELISA [19]. In contrast, in another study, 21% of positive sera for ANEA by IF did not react with nuclear envelope components using immunoblotting [20]. Generally, immunoblotting analysis showed that antibodies against proteins of the nuclear pore complex are associated with a more active and severe liver disease in PBC [3] or with an advanced state of PBC [4].

## Conclusion

Results of the present study indicate that efficient detection of ANEA may be obtained by indirect IF analysis of patients' sera using cells appropriately fixed with formaldehyde. In particular, mild fixation reduces considerably the staining of cytoplasmic or nucleoplasmic antigens in Hep2 and HeLa cells and permits a better resolution of nuclear membrane epitopes. In view of the potential diagnostic significance of ANEA in a number of rheumatic diseases, as well as in PBC, we consider that this method might be a valuable additional tool to the clinical laboratory practice.

## Competing interests

The author(s) declare that they have no competing interests.

## Authors' contributions

VT and ET: Acquisition and analysis of data

HP: Acquisition of data and technical supervision

APN and TA: Technical assistance and revising the manuscript

MK: Preparation of sera and follow-up of patients

EK: Interpretation of data and revising the manuscript

EC: Design of the study, interpretation of data and revising the manuscript

PAT: Conception and design of the study, analysis and interpretation of data, drafting and revising the manuscript

All of the authors read and approved the final manuscript.
